# Book Review

**Published:** 1939

**Authors:** 


### Reviews of Books

**Aequanimitas, with Other Addresses.** Third Edition. By Sir W. Osler, Bt., M.D., F.R.S. Pp. xiv., 453. London: H. K. Lewis & Co. Ltd. 1939. Price 7s. 6d.—It is always a pleasure to meet old friends, especially when the latter seem possessed of perennial youth. Most of Osler's addresses are well known to all senior members of the profession, and the advice that he gave to his colleagues and students twenty years ago is equally opportune to-day. In view of the ever-expanding medical curriculum his protest against mental indigestion among medical students applies with even greater force to the present day than when he spoke of it with such emphasis at the beginning of the century. All generations, and both sexes, can find help and guidance in this book, and one can only hope that Osler's interest in the philosophy of medicine and appreciation of good literature will be appreciated by those who are looking for something rather more inspiring than a contemporary text-book. Finally, this volume can be recommended as a fit companion to the small but select list of books that Osler himself recommends for bed-time reading and for the attainment of equanimity which under present conditions we need even more than in his day.

**Health in Relation to Occupation.** By H. M. Vernon, M.D. Pp. viii., 355. Illustrated. London: Oxford University Press (Humphrey Milford). 1939. Price 15s.—One of the characteristics of post-war medicine has been the growing realization that disease processes cannot be understood only at the bedside but must be viewed in the light of their home surroundings and occupational causes. It is probable that the sociologists and public health administrators have in many cases taken the lead where medicine has already pointed the way, but even so there has frequently been in the past a gulf between sociological and curative medicine that has been hard to bridge. Vernon's book is a very real attempt to link these two communities and in the process he undoubtedly covers a tremendous amount of ground. He has collected together figures that are ordinarily to be found only in Government Blue Books, and has presented them in a readily assimilable form, and in addition has surveyed to a large extent the outstanding research work that has been done in this country in the last twenty years. The book should prove of real value to all interested in social medicine, and it is to be hoped that medical students will find it of very definite value in appreciating the occupational aspects of their patients.

**Electric Excitation of Nerve.** By B. KATZ, M.D., Ph.D. Pp. ix., 151. London: Oxford University Press (Humphrey Milford). 1939. Price 10s. 6d.—This is an interesting review of the problems of the electric phenomena of nerve and nerve excitation as they have been dealt with by various authorities in recent years. It was compiled at the invitation of the editors of the *Ergebnisse der Physiologie*, but racial discrimination forced the prospective publisher in Berlin to refuse the manuscript. It found, however, in the Oxford University Press a publisher not influenced by Nazi pressure or considerations of pure Aryanism. The author expressly states that this is not a monograph embodying original work, but the author's own work in this field, and his familiarity with the work and theories of others have enabled him to present in a readable form an extremely valuable critical review of the experimental observations and theoretical interpretations of the events which follow the application of an electrical stimulus to a nerve and precede the propagation of an impulse.

**Textbook of Medical Treatment.** Edited by D. M. DUNLOP, M.D., F.R.C.P., L. S. P. DAVIDSON, M.D., M.R.C.P., and J. W. MCNEE, D.S.O., D.Sc., M.D., F.R.C.P. Pp. xx., 1,127. Illustrated. Edinburgh: E. & S. Livingstone. 1939. Price 25s.—This textbook of treatment, edited by Dunlop, Davidson and McNee, in the words of Professor A. J. Clark, " Provides a basis for the rational and critical judgment of modern therapeutic agents." There is no doubt that it fulfils these aims very adequately. The editors are helped in different sections by experts on their various subjects. A great need is felt for such a book as this when the introduction of new therapeutic agents is almost a daily occurrence. Many have been taken up with great enthusiasm, only to be dropped because of disappointment, perhaps only after short trials or inadequate dosage. This book sets out to show in great detail the limitations of this new therapy as well as its advantages. The general scheme of textbooks of Medicine is adhered to—that is to say, Infectious Diseases are dealt with first and the various systems are then treated in detail. Before each treatment is described, a short note on diagnosis is found, which is helpful and to the point. It is difficult to pick out special chapters of note where all are good. Professor Davidson, a recognized authority on blood diseases, writes an admirable survey on the treatment of anæmias and blood diseases. Professor McNee is no less at home on Diseases of the Liver and Gastro-Intestinal tract. Dr. A. R. Gilchrist and Dr. I. G. Hill describe in great detail and with exemplary clearness the treatment of Diseases of the Heart. There is an excellent chapter on Psychotherapy by Dr. MacCalman, which will be found of great use to the practitioner. Modern endocrine therapy, dietetics (including many diet sheets), vitamin deficiency and technical procedures are all clearly set out and described in this very easily readable, excellent textbook.

**Warwick & Tunstall's First Aid to the Injured and Sick.** Seventeenth Edition. Edited by F. C. NICHOLS, M.C., M.B., Ch.B., M.R.C.S., L.R.C.P., L.D.S. Pp. xv., 328. Illustrated. Bristol: John Wright & Sons Ltd. 1939. Price 2s. 6d.—The chief alteration in the seventeenth edition of Warwick & Tunstall's "First Aid" is the much fuller account given of Gas Poisoning in Warfare. The information in the various official A.R.P. handbooks is brought together into a uniform and continuous whole, upon which the editor is to be heartily congratulated. It is far easier to read in this form than scattered through the (at least) three official handbooks. There is also a new section on the fitting, use and care of the civilian respirator. Warwick & Tunstall continues to hold the field against its rivals as the best first-aid manual. In these days when every home is threatened, at any rate from the air, this is a book to read and to keep handy.

**Radiotherapy in Sinusitis.** By W. ANNANDALE TROUP, M.C., M.D. Pp. v., 51. Illustrated. London: Actinic Press Ltd. 1939. Price 3s. 6d.—It is only recently that the value of radiotherapy in cases of sinusitis has been widely recognized. In this book the author gives an admirable description of the apparatus and methods which he uses. There are useful chapters on the physiological action and application of infra-red and ultra-violet radiations, but ultra short wave, which has been used so extensively in recent years, receives only very short notice. A number of contra-indications are mentioned, but there is very little indication of what types of cases the author considers suitable for radio-therapeutic treatment, and apart from the description of some individual cases there is little indication of what results should be expected. There are a few very clear illustrations of the apparatus used, and there is a full index and good bibliography. This book should encourage the radiotherapist and rhinologist to make more use of a very promising method of treatment.

**Diathermy.** By W. BEAUMONT, M.R.C.S., L.R.C.P. Pp. x., 303. Illustrated. London: H. K. Lewis & Co. Ltd. 1939. Price 10s. 6d.—This book has as its main object the presentation, in language as simple as possible, of the fundamental principles and technique of treatment by means of high-frequency oscillating current in its different forms. These include diathermy, epithermy (of which violet rays are an example), inductothermy, the use of an ultra-high-frequency oscillating current, and short-wave diathermy. The book is well produced and printed, and the illustrations are excellent; they include good diagrams of electrical circuits, and also anatomical diagrams showing the placing of electrodes. There is a series of questions at the end of each chapter which may be of use to students, and at the end of the book there is a glossary of technical terms and a good index.

**Infra-Red Irradiation.** Second Edition. By W. BEAUMONT, M.R.C.S., L.R.C.P. Pp. xii., 159. Illustrated. London: H. K. Lewis & Co. Ltd. 1939. Price 6s. 6d.—This is the second edition of a book much used by students in electrical and massage departments. It deals with the physics, physiology, apparatus, technique and clinical aspect of the subject. In addition an anlaysis of over 1,000 unselected cases is given at the end of the book. It is well printed, illustrated and produced. That portion of the book given up to the physics and theory shows how much of this is purely speculative and might with value be condensed still further. Whilst those who use infra-red and radiant heat feel that certain types of case respond to the one and not to the other, the author puts a warning. He says: "The question of depth of penetration of the actual rays looms far too large at the present time. It is fostered in all probability by the desire of manufacturers to find some peg on which to hang their claims for superiority of apparatus." In the beginning of the chapter on treatment, he reminds the reader of the necessity of thoroughly investigating a case before commencing to treat the symptoms. He then points out that the treatment of symptoms forms a very large part of the practice of medicine, and, further, is in many cases the most that the practitioner can hope to achieve.

**Surgical Diagnosis.** By S. POWER, M.S., F.R.C.S. Pp. vii., 228. Illustrated. Bristol: John Wright & Sons Ltd. 1939. Price 12s. 6d.—This book certainly gives a great deal of information not to be found elsewhere. In the earlier pages the reader is told, among other things, of muscle spasm keeping bone ends apart in tuberculous joint disease, and of the epiphysis slipping upwards in Coxa Vara. On page 50 he finds an entirely new version of that classical diagram, the synovial sheaths of the palm, differing in almost every respect from that commonly accepted by anatomists and surgeons. It will also interest our reader to learn that Raynaud's Disease affects chiefly the lower extremities. This on page 53, if he gets as far. Somehow we do not think he will.

**Rectal Surgery.** By W. ERNEST MILES, F.R.C.S., F.A.C.S. Pp. xi., 359. Illustrated. London: Cassell & Co. Ltd. 1939. Price 17s. 6d.—This is a book which will interest both the surgeon and the general practitioner—the former from the fact that it is a record of the interpretation of clinical findings and the methods of dealing with these adopted by the author, a surgeon of wide experience in this speciality who has not followed the beaten track, but whose initiative, observation, and imagination have led him to evoke new lines of treatment, based on anatomical and clinical investigation, operative experience, and the results of this, gained in a clinic devoted to rectal surgery. The anatomy of the important structures in this region is amply detailed, and, what is of more value, the surgical importance of these is clearly explained. The general practitioner will appreciate this work for its many and illuminating diagrams, which support and illustrate the text, and for the logical explanations of the clinical pictures and the reasoned lines of treatment that these lead up to, which should tend to eliminate the application of a single method to lesions having different origins and therefore needing different treatment. The emphasis laid on the wisdom of radical operation, in hands capable of this, for cancer of the rectum, as elsewhere, will be appreciated by the surgeon, rather than attempts to save the patient from a permanent colotomy by a less radical procedure. The matter is up to date and sound, the illustrations are well chosen and clearly depict what they are meant to. As a monograph of the author's own experience and original work, references are naturally few, but the index is ample.

**Diverticula and Diverticulitis of the Intestine.** By H. C. EDWARDS, M.S., F.R.C.S. Pp. xii., 335. Illustrated. Bristol: John Wright & Sons Ltd. 1939. Price 25s.—This monograph, as Mr. Edwards explains in his preface, represents the Jacksonian Prize Essay of 1932 revised and brought up to date. The essay was an excellent piece of work and it deserves the greater publicity which it should now obtain. The author goes most thoroughly into every aspect of congenital diverticula first of all, and then into that of acquired diverticula. He has got together a wealth of material from many sources and his work is a mine of information. Diverticula of the large bowel are of the greatest general interest: there can be few medical men who are not called upon from time to time to diagnose and treat some pathological condition arising from diverticulitis of the colon. There are so many of these complications that the author's careful analysis of the methods of dealing with each and of the results obtained must prove extremely helpful. There seems to be no mention of the acute pneumoperitoneum which sometimes follows a rupture of a diverticulum. Though not common, it is a complication which should be recognized, for an early operation before general peritonitis has been established may save the patient's life. The general "get up" of the book, its printing, illustrations and indexing, leave nothing to be desired and one cannot fail to find the text of the utmost interest.

**Orthopaedic Appliances.** By H. H. JORDAN, M.D. Pp. xxii., 412. Illustrated. London: Oxford University Press (Humphrey Milford). 1939. Price 21s.—This book fills a gap which has been left by other books on this subject. It gives a complete survey of all types of splints, especially the more expensive splints. Illustrations are given of splints used for the correction of deformities and for post-traumatic wear. The illustrations are excellent and the text goes into the minutest detail, so that the manufacturer should have no difficulty in copying them. The only criticism we have to make is that the splints are so complicated and costly that they cannot be considered for hospital use. It would be of great use for orthopædic surgeons, especially those connected with the Ministry of Pensions, and surgical instrument makers.

